# Alien Phytogeographic Regions of Southern Africa: Numerical Classification, Possible Drivers, and Regional Threats

**DOI:** 10.1371/journal.pone.0036269

**Published:** 2012-05-04

**Authors:** Sanet Hugo, Berndt J. Van Rensburg, Abraham E. Van Wyk, Yolande Steenkamp

**Affiliations:** 1 Department of Zoology and Entomology, DST-NRF Centre of Excellence for Invasion Biology, University of Pretoria, Pretoria, South Africa; 2 School of Biological Sciences, University of Queensland, Brisbane, Australia; 3 Department of Plant Science, University of Pretoria, Pretoria, South Africa; 4 Biosystematics Research & Biodiversity Collections Division, South African National Biodiversity Institute, Pretoria, South Africa; Lakehead University, Canada

## Abstract

The distributions of naturalised alien plant species that have invaded natural or semi-natural habitat are often geographically restricted by the environmental conditions in their new range, implying that alien species with similar environmental requirements and tolerances may form assemblages and characterise particular areas. The aim of this study was to use objective numerical techniques to reveal any possible alien phytogeographic regions (i.e. geographic areas with characteristic alien plant assemblages) in southern Africa. Quarter degree resolution presence records of naturalised alien plant species of South Africa, Lesotho, Swaziland, Namibia and Botswana were analysed through a divisive hierarchical classification technique, and the output was plotted on maps for further interpretation. The analyses revealed two main alien phytogeographic regions that could be subdivided into eight lower level phytogeographic regions. Along with knowledge of the environmental requirements of the characteristic species and supported by further statistical analyses, we hypothesised on the main drivers of alien phytogeographic regions, and suggest that environmental features such as climate and associated biomes were most important, followed by human activities that modify climatic and vegetation features, such as irrigation and agriculture. Most of the characteristic species are not currently well-known as invasive plant species, but many may have potential to become troublesome in the future. Considering the possibility of biotic homogenization, these findings have implications for predicting the characteristics of the plant assemblages of the future. However, the relatively low quality of the dataset necessitates further more in-depth studies with improved data before the findings could be directly beneficial for management.

## Introduction

Broad-scale patterns of spatial variation in biotic diversity have received the attention of ecologists and biogeographers for almost two centuries [1]–[4]. More recently, improved methods of statistical analysis, coupled with powerful computer programs have enabled more objective macroecology and biogeography studies [5]. For example, macroecology and biogeography have recently been employed in spatial conservation planning studies, to indicate the regions where conservation efforts should be maximised (e.g. species rich areas) as well as the regions that are threatened by human-activities that are detrimental to biodiversity [4], [6]–[8]. One of these threats is the deliberate or accidental human-facilitated introduction of species into regions where they do not occur naturally [8]. Although most introduced or alien species do not form viable populations in the introduced range, many have become naturalised in the new environment and some are able to invade natural or semi-natural ecosystems, often causing loss of natural biodiversity, water shortages, loss of crop and forest production, and increased soil erosion [9]–[13].

Management intervention of harmful alien species is expensive, often labour intensive, and not always effective [14]–[16]. To reduce cost and increase efficiency it is necessary to identify priority areas and species on which to focus research and control efforts [9]–[12]. To this end, researchers have attempted to track the ability of certain alien species to invade areas (e.g. predictions of future spatial range, through niche modelling methods), the potential for invasive species to transform their introduced habitat, or the vulnerability of certain areas to invasion [9]–[11], [17]–[20]. However, with hundreds of naturalised alien species recorded from southern Africa for example [10], [19], [21], timely identification and control of each potentially harmful invasive species seems to be a futile task. Several South African reviews delineated whole sets of priority species, by listing and describing alien species that are currently of most concern (i.e. with known economical and ecological impacts), often organising these species lists according to the biomes or occasionally the political provinces in which they are most troublesome [12], [15], [19], [22]. Conservation actions are often undertaken at a provincial level thereby justifying the organisation of alien species into political provinces; however it makes little sense ecologically, to impose artificial boundaries on naturally established species distributions. In this study, by ‘natural establishment’, we mean the spontaneous colonisation of alien plants in response to natural environmental conditions (which may or may not be spatially coincident with the biomes) as well as human-caused conditions/transformations, but without deliberate aid by humans [21], [23].

Naturalised alien species may adapt successfully to a new set of environmental conditions [24] and many invasive alien species are widespread and present in a range of habitats (e.g. *Acacia mearnsii* in southern Africa) [19]. Nevertheless, the spatial distributions of naturalised and invasive alien plants are generally constrained by environmental factors similar to those constraining native plant species. Studies have shown that the distribution of such species in the introduced range (adventive range) is often a reflection of the prevalent environmental conditions in their native range [12], [17]–[19], [21], [25]–[27]. For example, in southern Africa species of *Prosopis* are prominent invaders of arid areas [28]. Consequently, we may expect to find that groups of alien plants with similar environmental requirements and tolerances are associated with specific areas, thereby forming assemblages of alien plant species that characterise those areas [19], [28].

The possibility that naturally established alien species assemblages could exist in the introduced range encourages much further research (e.g. on their spatial distributions, characteristics and determinants) and we propose that such assemblages could be prioritised for invasive species management. In this regard, previous studies in South Africa, by Richardson and colleagues in 2004 [29] and Thuiller and colleagues in 2006 [30], explored the link between the shared traits of successful alien plant species and their spatial distributions. They used classification analysis to describe clusters of invasive alien plant species as species assemblages with ecologically meaningful spatial distributions, and revealed some intrinsic and extrinsic factors that determine the invasive potential of alien species in those particular geographic areas where their niche requirements are met [29], [30]. For both studies, the clusters could not share species although the spatial distributions of the clusters may overlap geographically [29], [30]. What has not yet been considered before, at least for southern Africa, is whether the study area itself may be spatially partitioned into non-overlapping geographical areas characterised by their alien plant species compositions, which might serve as more ecologically sensible alien species management districts than political provinces. However, before such a venture could be considered, we need to first find and describe such regions, if they exist.

Non-overlapping geographic regions characterised by distinct floristic compositions are termed phytogeographic regions [31]. This method of classifying different regions according to their species compositions has a long history with regard to endemic plant species of Africa [31]–[34]; however, to our knowledge, alien plant species have not been studied in this way. The designation of phytogeographic regions is often based on expert opinion, for example, Van Wyk and Smith [32] relied on expert opinion to designate the Cape Floristic Region, the Succulent Karoo Region, and the Maputaland-Pondoland Region as larger phytogeographic regions in southern Africa encompassing a series of local centres of endemism (see also [33]). However, more objective methods of numerical analysis are available, one of which was successfully used by Van Rooy and Van Wyk [35] on the moss flora of southern Africa, and by Steenkamp and colleagues [31] on the native plant genera of southern Africa (see also [34]). In these numerical studies a grid is applied to the analysed geographical area, and therefore the term ‘phytogeographic region’ would then be defined as a group of grid cells of similar floristic composition [31]. Endemic phytogeographic regions are often evaluated with regard to broad-scale current and prehistoric climatic and geological factors that have likely formed the endemic plant assemblages over the long term [31], [32], [34]. In contrast, the adventive spatial ranges of alien plant species are probably shaped by recent or current environmental and human-related factors that were prevalent during and after the introduction of these species.

The aim of this study was to reveal ecologically meaningful phytogeographic regions of alien plant species in southern Africa, by means of numerical classification analysis. We then hypothesise on the possible drivers of these regions, and discuss their implications for alien species management and research in the future. We considered only naturalised and invasive alien plant species – casual introduced species were not included in the data. Throughout the article, we use the terms ‘naturalised’ and ‘invasive’ in accordance with the definitions in Richardson and colleagues’ 2000 paper [36].

## Methods

### Study Area and Data

The data we used were records of all naturalised alien plant species from the National Herbarium, Pretoria Computerised Information System (PRECIS), for Namibia, Botswana, South Africa, Lesotho and Swaziland (Table S1). Distribution records in PRECIS are currently most complete for these southern African countries [31] and of these, South Africa, Lesotho and Swaziland provides the best data. Analyses were conducted at the quarter degree square resolution because PRECIS data is mainly available at this resolution. The species rank was the lowest taxonomic level which was analysed: records of different infraspecific taxa were pooled and hybrids were not considered. Ultimately, 861 alien plant species were included in the analysis.

**Figure 1:**
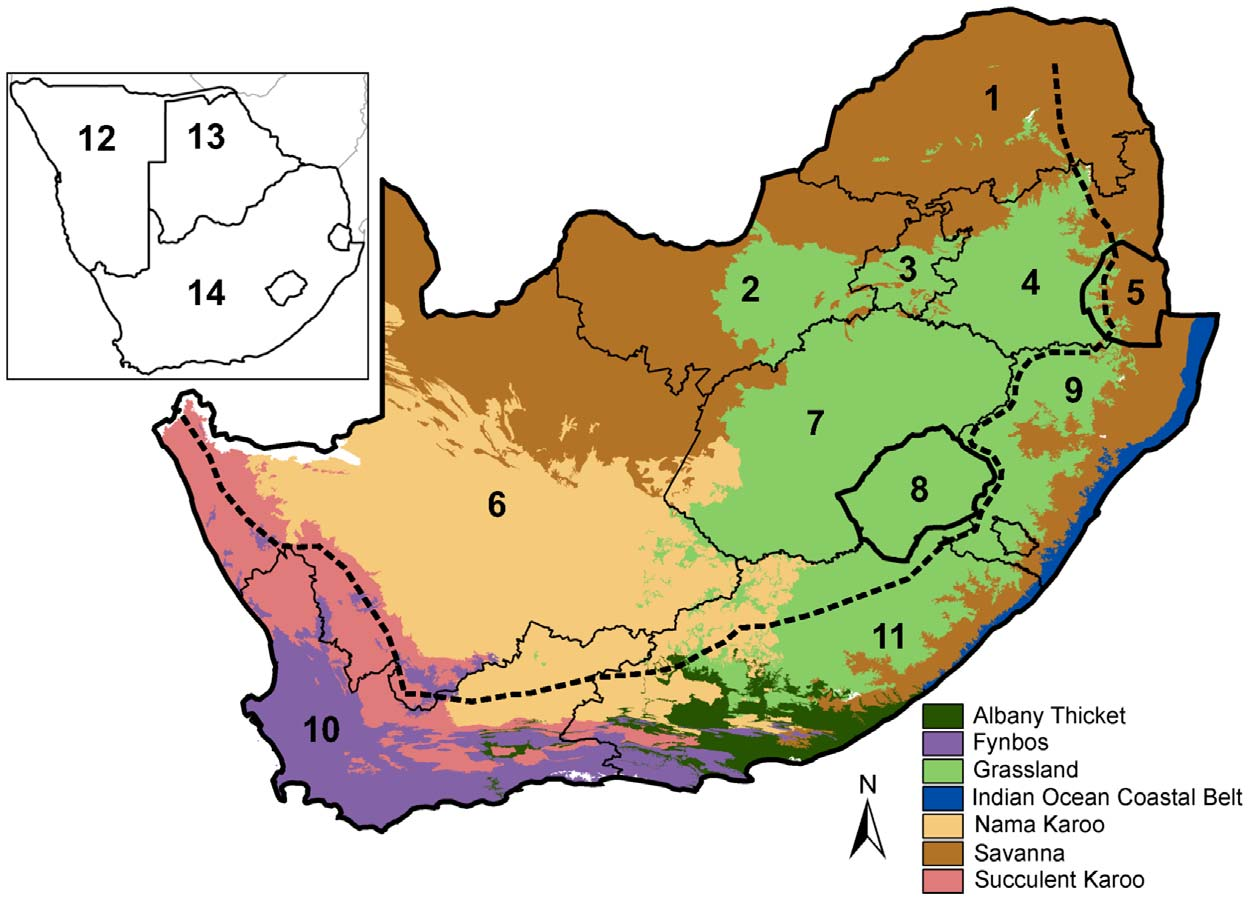
The main spatial features of the study area, focusing on South Africa, Lesotho and Swaziland. Here we represent the political boundaries of the general study area (i.e. including Namibia and Botswana) and the spatial distributions of seven of the biomes in South Africa, Lesotho and Swaziland, as based on the biome classifications of Mucina & Rutherford [37]. The forest and desert biomes occupy very little of South Africa’s surface area, and are not shown here. The approximate position of the Great Escarpment in South Africa is represented by the dashed line. The numbers indicate the following political regions: 1. Limpopo Province, 2. North West Province, 3. Gauteng Province, 4. Mpumalanga Province, 5. Swaziland, 6. Northern Cape Province, 7. Free State Province, 8. Lesotho, 9. KwaZulu-Natal Province, 10. Western Cape Province, 11. Eastern Cape Province. The insert shows the countries 12. Namibia, 13. Botswana, and 14. South Africa.

Southern Africa is a geologically and climatically diverse region. Notable drivers of floristic composition are the topographical and geological variation between the interior plateau of relatively high altitude, that is bordered on three sides by mountain ranges forming the Great Escarpment, beyond which is a sharp drop in altitude towards the coastal plains (Figure 1) [31], [37]. Notable climatic drivers include a strong moisture gradient from arid regions in the west to humid regions in the east, and a variation in the seasonality of rainfall, from summer rainfall comprising most of southern Africa, to a smaller winter rainfall area in the west, and year-round rainfall on the south-west coast between the winter and summer rainfall areas [31], [37]. Finally, eight biomes have been described for South Africa, the Grassland, Savanna, Albany Thicket, Nama-Karoo, Succulent Karoo, Forest, Fynbos and Indian Ocean Coastal Belt (Figure 1) [37]. The Nama-Karoo, Succulent Karoo and Savanna biomes continue north into Namibia, which also includes the Desert biome [32], [38]. Botswana is mainly covered by Savanna biome with a smaller area of Nama-Karoo [32], [39].

### Analyses

The presence and spatial distributions of discernable phytogeographic regions in the study area were assessed based on a divisive hierarchical classification technique [31]. Grid cells were grouped into clusters based on the combination of alien plant species present in each cell as recorded in the PRECIS dataset. The statistical program TWINSPAN (two-way indicator species analysis) [40] was used to conduct this classification. The dataset was converted to a binary presence/absence data matrix and then inputted, via the programs Turboveg 1.97 (International Single User Version, Stephen Hennekens) and Megatab 2.2 (Elsware), into TWINSPAN.

Despite criticisms directed at TWINSPAN, it is still widely used and has been shown by many previous studies from many parts of the world to be robust, effective, relatively objective, and successful in distinguishing geographic areas with characteristic assemblages of species or higher taxa [31], [35], [41]–[45]. Also, TWINSPAN is particularly suitable for datasets that are complex, large and noisy [31], [35], [41], [45]. This is essentially the state of the PRECIS dataset, which, as with many species atlas datasets, contains many gaps in terms of locations for which no records are available or for which the available data are not particularly reliable or representative [21], [31], [35].

The successive clusters of grid cells derived from the initial dendrogram provided by TWINSPAN were depicted on maps representing the study area (ArcView GIS 3.3, ESRI Inc. 2002). We report only those clusters of grid cells that we considered to be ecologically meaningful with the potential for further interpretation. Successive divisions in TWINSPAN were continued until no further interpretable or meaningful geographic regions could be identified. Clusters at any level of division in the hierarchical classification analysis that were too small or too randomly spaced across the study area to allow for meaningful interpretation were disregarded; however such clusters altogether comprised only a small part of the study area.

To select environmental factors that we considered to be possible drivers of these phytogeographic regions, we subjectively assessed the discernable phytogeographic regions as plotted on the maps, together with knowledge of the spatial distributions of environmental and human factors known to drive plant species distribution at the spatial scale studied and at the recent timescales governing alien species [21], [32]. Additional insight is provided by knowledge of the environmental requirements of the alien species that characterise each phytogeographic region. “Characteristic” species were considered to be those whose spatial distributions coincide more with specific phytogeographic regions than with the rest of the study area (corrected for the size of the region), and that therefore likely contributed most to TWINSPAN’s classification of grid cells into clusters. Some of these species are mentioned in the discussion; more complete lists of the characteristic species of all phytogeographic regions are provided in Table S2.

We relied mainly on subjective expert opinion to refine and interpret the output of the TWINSPAN analysis (see White’s 1993 paper [33] and Van Wyk and Smith’s 2001 publication [32] for discussions of the benefits of this method); however, we based these interpretations on a wealth of published and mapped information on the areas and species studied. We still consider the TWINSPAN method to be more objective than relying solely on expert opinion for the demarcation of phytogeographic regions, as none of the alien phytogeographic regions described here were expected *a priori*. Nevertheless, although Namibia and Botswana contain too few datapoints to justify further analysis, South Africa, Lesotho and Swaziland, for which better PRECIS data coverage and spatial environmental data are available, were further examined through calculations of percentage overlap and statistical tests to support our inferences of the possible environmental drivers of alien phytogeographic regions.

Mucina and Rutherford published geographic information system (GIS) maps in 2006 [37] depicting the spatial distributions of the biomes in South Africa, Lesotho and Swaziland. To convert these biome GIS maps to a quarter degree resolution that may be compared with the various phytogeographic regions, we assigned each quarter degree grid cell from the PRECIS dataset to a particular biome if more than 50% of the area of the grid cell is overlapped by that biome. Grid cells that were not more than 50% covered by any single biome (i.e. could not be assigned to any biome) were disregarded as they were few in number and unlikely to have a notable influence on the results. We then estimated and ranked the relative importance of each of the biomes in the various phytogeographic regions by calculating for each phytogeographic region the percentage of its grid cells that were assigned to each particular biome. The Forest and Desert biomes were excluded, as these biomes occupied very little or none of the surface area of South Africa, Lesotho or Swaziland.

The spatial distribution of each individual phytogeographic region was further analysed using the SAS version 9.2 (SAS Institute Inc. 2008) procedure ‘PROC LOGISTIC’, which is a logistic regression procedure that allows the use of presence-absence (i.e. binary) data to model the probability that a grid cell belonging to a particular phytogeographic region coincides spatially with selected environmental factors [46]. Based on our subjective interpretation as explained previously and after conducting tolerance tests for collinearity [46], [47] we selected mean annual precipitation, mean monthly maximum and minimum temperatures for the hottest and coldest months respectively, and the percentage of the surface area of each grid cell that is cultivated, degraded or irrigated, as predictors of phytogeographic regions. All of these factors were represented at a quarter degree resolution. Precipitation and mean monthly maximum and minimum temperatures were calculated from monthly data based on interpolated climate surfaces for the past 30–50 years, and supplied by the South African Computing Centre for Water Research [48]. Cultivated area and degraded area were from the National Land Cover Database as captured by Landsat TM satellite imagery mainly between 1994 and 1995 [49]. A spatial distribution map of irrigated areas for South Africa was published by the Agricultural Research Council – Institute for Soil, Climate, and Water (2000) and was downloaded at the Agricultural Geo-Referenced Information System (AGIS) website (www.agis.agric.za).

In some cases, certain predictors had nonlinear correlations with the response variable and this was revealed if the inclusion of the square terms of those predictor variables significantly improved the model [46]. In some of the models, a log transformation was applied to certain predictors to improve heteroscedasticity [46]. To test which combination of predictor variables best explain variation in the response variable (i.e. which model is best), ‘PROC LOGISTIC’ supplies Akaike’s information criterion (AIC) values, of which smaller (or more negative) values indicate a better model. AIC values do not mean anything by themselves and are used to compare models with different predictor variables and the same response variable to select the best available model. Thus, AIC values could not be used to compare different subsets of a dataset, i.e. different phytogeographic regions, and were not reported. To indicate the amount of variation in the response variables that is explained by the predictor variables of the ‘best’ models, we report Max-rescaled R-square values that are appropriate for logistic regression (see [50] for an explanation of this adjusted R-square value).

## Results

**Figure 2:**
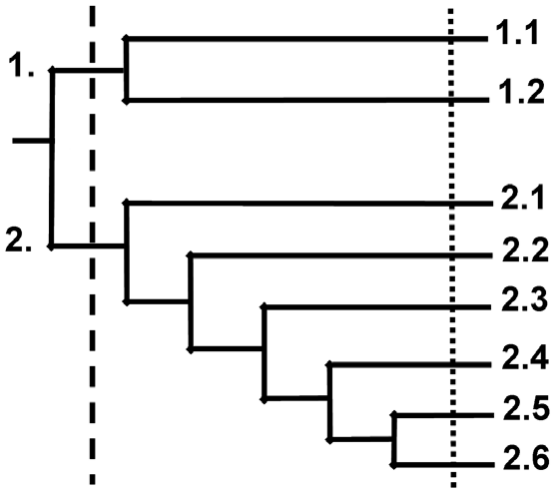
The dendrogram of the TWINSPAN classification analysis showing two levels of division. The initial TWINSPAN results was a dendrogram, which is represented here in a simplified form showing only the ecologically meaningful clusters (i.e. phytogeographic regions) from two levels of division (– – – – higher level; ▪▪▪▪▪ lower level). These phytogeographic regions were: 1. the Greater Arid Region, which includes the 1.1 Arid, and 1.2 Orange River regions; and 2. the Multiclimate Region, which includes the 2.1 Escarpment, 2.2 Northern, 2.3 Agricultural, 2.4 Western Cape, 2.5 Grassland, and 2.6 Savanna Regions.

**Figure 3:**
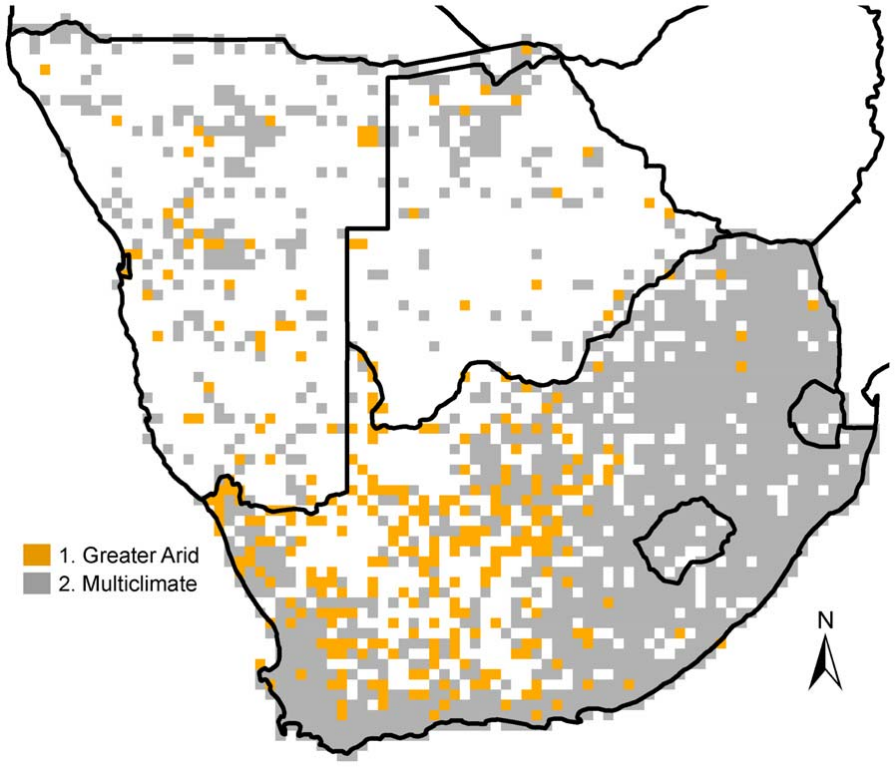
Spatial distributions of the higher level phytogeographic regions. The grid cells comprising the two main ecologically meaningful clusters as derived from the TWINSPAN cluster analysis (see Figure 2), were plotted on maps to indicate the spatial distributions of the 1. Greater Arid and 2. Multiclimate phytogeographic regions.

The meaningful clusters resulting from successive divisions of the presence records of naturalised alien plant species were depicted in a dendrogram (Figure 2), and the clusters of two levels of division in this dendrogram were chosen to be depicted on the maps in Figures 3 (higher level of division) and 4 (lower level).

Here we summarise the process of division in the order in which the clusters of grid cells forming phytogeographic regions became separated from the main dataset. See the Discussion section for more information on the names assigned to the phytogeographic regions (see also the spatial features depicted in Figure 1). The first meaningful division by TWINSPAN (i.e. the higher level division) showed two clusters that could be defined as phytogeographic regions (Figures 2 and 3), and that could be assumed to be the regions that differed most strongly from one another in terms of their characteristic alien plant species compositions (Table S2). The larger of the two regions, the Multiclimate phytogeographic region, was spread over the entire study area (Figure 3) and surrounded a smaller phytogeographic region, the Greater Arid region.

**Figure 4:**
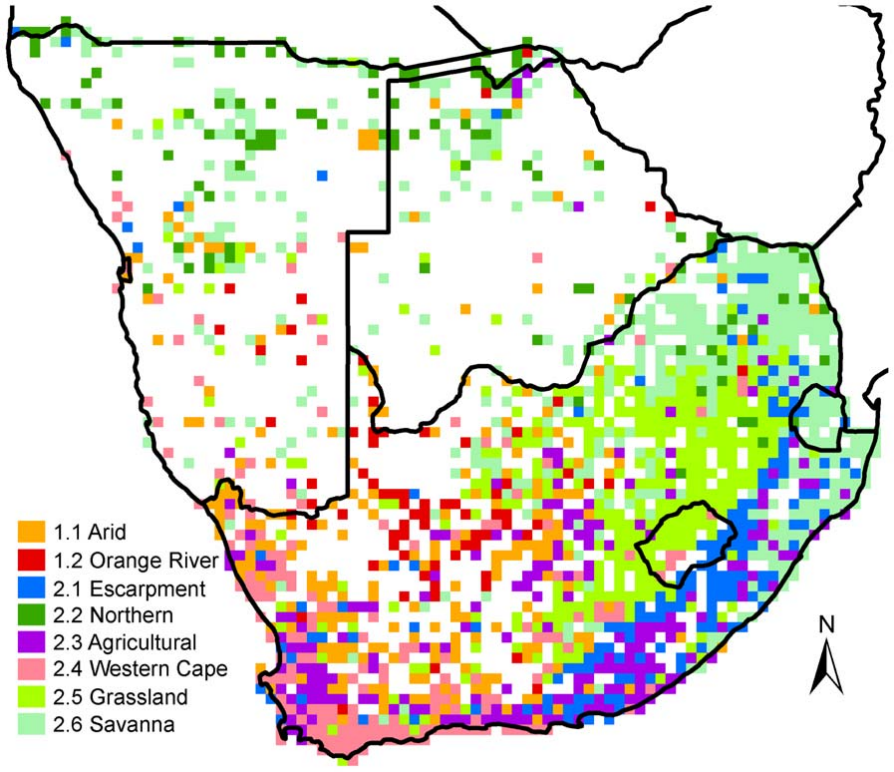
Spatial distributions of the lower level phytogeographic regions. As in Figure 3, the grid cells comprising the lower level phytogeographic regions as derived from the cluster analysis (Figure 2) are depicted: the 1.1 Arid and the 1.2 Orange River phytogeographic regions were subdivided from the Greater Arid region, and the 2.1 Escarpment, 2.2 Northern, 2.3 Agricultural, 2.4 Western Cape, 2.5 Grassland and 2.6 Savanna regions were subdivided from the Multiclimate region.

After further subdividing the Greater Arid region, two more phytogeographic regions, the Arid region and the Orange River region, were revealed (Figures 2 and 4). After subdivision of the Multiclimate region, six more phytogeographic regions were revealed: the Escarpment region, followed by the Northern region, the Agricultural region, the Western Cape region, and finally, the Grassland and Savanna regions (Figures 2 and 4). Any further subdivisions of these eight lower level phytogeographic regions yielded small, vague, spread-out clusters that likely represent noise. Therefore, no further subdivisions are reported.

Table 1 lists the percentages of grid cells of each phytogeographic region that were assigned to each biome in South Africa, Lesotho and Swaziland. The Greater Arid phytogeographic region spatially coincided mainly with the arid Nama-Karoo and Succulent Karoo biomes, although arid parts of the Savanna biome also made a large contribution especially within the lower level Orange River region. Within the Multiclimate phytogeographic region, *ca*. 68% of the Grassland phytogeographic region coincided with the Grassland biome, and *ca*. 71% of the Savanna phytogeographic region coincided with the Savanna biome. Around 50% of the Escarpment phytogeographic region coincided with the Grassland biome and *ca*. 20% with the Savanna biome. Further, nearly 50% of the Western Cape phytogeographic region coincided with the Fynbos biome, with large contributions also made by the Succulent Karoo (*ca*. 20%) and the Nama-Karoo (*ca*. 15%). Several biomes were prominent in the Agricultural phytogeographic region, most notably the Grassland (almost 30% of the Agricultural phytogeographic region) and Fynbos (more than 20%) biomes. The Northern region was not analysed as only a few scattered outliers are present in South Africa, Lesotho and Swaziland.

**Table 1:** The percentage of grid cells of each phytogeographic region that has been assigned to each particular biome [37] (see the Methods section for more details).

	Albany Thicket	Indian ocean coastal belt	Grassland	Savanna	Fynbos	Succulent Karoo	Nama-Karoo
1. Greater Arid	1.25	0.42	6.67	20.83	5.83	18.75	42.92
1.1 Arid	1.12	0.56	7.82	16.76	7.26	24.58	37.99
1.2 Orange River	1.67	1.67	3.33	31.67	1.67	1.67	58.33
2. Multiclimate	3.45	3.45	35.77	31.82	11.87	5.13	8.16
2.1 Escarpment	8.50	3.92	50.98	21.57	7.84	3.92	3.27
2.2 Agricultural	11.46	3.13	28.65	11.98	23.44	7.29	14.06
2.3 Western Cape	1.92	0.00	8.97	4.49	47.44	19.87	15.38
2.4 Grassland	0.64	0.64	68.17	17.04	2.25	1.61	9.97
2.5 Savanna	0.31	8.90	15.95	71.17	0.61	0.92	2.15

Table 2 shows, for each phytogeographic region, the combination of environmental and human variables that best predicted the spatial distribution of that phytogeographic region (i.e. the best logistic regression model) together with the Max-rescaled R-square (*R* ^2^) values. Overall, the natural environmental factors (precipitation and mean maximum and minimum monthly temperatures) seemed to be more important than the human-caused factors (irrigated, cultivated and degraded area) in that they were more often included into the best models and also had greater Chi-square values (Table 2). Between 22% and 47% of variation in the response variables (i.e. distribution of phytogeographic regions) was explained by the relevant predictors in the reported models, except for the Agricultural region where only 9% was explained (see the *R* ^2^ values in Table 2). Judging by this, it is likely that the phytogeographic regions are partly determined by factors that we have not considered in the current study. The ‘best’ models of individual phytogeographic regions are further discussed in the discussion section.

**Table 2:** The Chi-square values, significance levels^a^, and Max-rescaled R-square values (*R^2^*, see Nagelkerke 1991) of natural and human-caused factors that were included in the logistic regression models that best explained (in terms of probability of co-occurrence) the spatial distribution of each phytogeographic region.

	Irrigated area	Cultivated area	Degraded area	Maximum temperature	Minimum temperature	Precipitation	*R^2^*
1. Greater Arid	n.i.^b^	n.i.	n.i.	****33.90	^†††^13.35	^††††^159.16	0.47
1.1 Orange River	n.i.	*5.48	n.i.	****40.25	^†††^11.04	^††††^26.15	0.39
1.2 Arid	n.i.	n.i.	n.i.	n.i.	n.i.	^††††^156.55	0.33
2. Multiclimate	n.i.	n.i.	n.i.	^††††^33.90	***13.35	****159.16	0.47
2.1 Escarpment	*4.08 L	^††††^17.43	****22.81 sq	^††††^54.05 L	****20.17 sq	*4.54 L	0.26
2.2 Agricultural	*5.81 L	n.i.	n.i.	****21.65	n.i.	n.i.	0.09
2.3 Western Cape	n.i.	^††^7.25	n.i.	^††††^60.64	*6.64	****26.26	0.22
2.4 Grassland	^††^9.96	*5.61 L	^††^7.83	**8.61 L, sq	^††††^116.78	****25.27 sq	0.39
2.5 Savanna	n.i.	n.i.	^†^5.30	****18.46	****23.78	****56.18	0.47

a Significance levels: positive, * P<0.05; ** P<0.01; *** P<0.001; **** P<0.0001; negative, ^††^ P<0.01; ^†††^ P<0.001; ^††††^ P<0.0001.

b Abbreviations: n.i., not included in the model; L, log of predictor used; sq, square term of predictor included (nonlinear relationship).

## Discussion

Several alien plant phytogeographic regions were identified based on the PRECIS data, which we considered ecologically meaningful based on their spatial associations with various habitat, climatic and human-related factors. At the lowest meaningful level of division, a total of eight alien phytogeographic regions were revealed, two of which were subdivided from the Greater Arid phytogeographic region, and six from the Multiclimate phytogeographic region.

Our subjective interpretations of the determinants of these phytogeographic regions, as discussed further on in the article, were generally supported by further statistical analyses. Compared to human-related factors, natural environmental factors were generally more important predictors of the spatial distributions of alien phytogeographic regions in logistic regression models. It is possible that the alien species that characterise the various phytogeographic regions (Table S2) are less dependent on human activities because they are invasive species that thrive in the natural habitat of their introduced range [21]. However, this contrasts with previous studies stressing the importance of human activities for explaining invasive alien plant spatial distributions [29], [30]. Other possible reasons for the weaker influence of human-related factors in the current study are that these variables might be more important at finer spatial resolutions than that of the current study (scale-dependent effects) [21], or that the phytogeographic regions might be associated with human activities that had not been considered in the current study.

Other atlases of the geographic distribution of alien plants are available for at least parts of the study area, most notably the Southern African Plant Invaders Atlas, or SAPIA, database; however, this database is based on sight records of easily visible species along roads and as such is particularly biased towards larger woody species and human-disturbed environments [19]. Considering that we were more interested in the natural spread of *all* naturalised alien plant species, the SAPIA database is less suitable for the study because it introduces those biases that we would most like to avoid (but see [29], [30]). Therefore, we considered the PRECIS dataset more suitable, as it was based on specimen collection of all plants regardless of invasive status and growth form, more randomly placed with regard to natural and human-made landscape features, and has been shown to be useful for large-scale spatial numerical classification studies [31].

### Greater Arid Phytogeographic Region

The Greater Arid phytogeographic region (Figure 2) is associated mainly with hot, low rainfall areas (Table 2), like the arid and semi-arid Nama-Karoo biome and Kalahari regions in the west of South Africa and in Namibia, extending further west into the Succulent Karoo biome (Table 1; Figures 1 and 3)[37]–[39]. In accordance with Milton and Dean’s [28] survey in arid and semi-arid regions of South Africa, the Greater Arid phytogeographic region is characterised by arid-adapted taxa such as *Atriplex* spp., *Prosopis* spp., *Verbesina encelioides* and *Salsola kali*. The native ranges of these species are generally tropical or subtropical arid regions, especially in South and Central America and Australia [51]–[53].

The lower level Arid phytogeographic region spans the Greater Arid region, encloses the Orange River region, and closely matches the Greater Arid region in terms of characteristic species and biomes, except that species of *Prosopis* are much less important in the Arid region (Table 1; Figures 1, 3 and 4). The Orange River region is found on the border between the Savanna and Nama-Karoo biomes where these biomes are separated by the Orange River (Table 1; Figures 1 and 4) [37]. In contrast to the Arid region, which is characterised mainly by *Atriplex* and *Salsola* species, we consider *Prosopis glandulosa*, or honey mesquite, to be the most significant characteristic species of the Orange River region (present in 59% of the grid cells of this region), followed by *P. velutina*, or velvet mesquite (present in 52% of grid cells). Less important, but still noteworthy, are *Persicaria limbata* (third most characteristic species) and *Prosopis chilensis* (fourth most characteristic). These four characteristic species commonly colonise the water edge and the banks of permanent or temporary rivers or dry riverbeds, and are probably dependent on the Orange River and other water sources such as irrigation dams. Therefore, we suggest that permanent and temporary sources of water are essential drivers of the alien species found in the Orange River region, although irrigated area is not included in the best model for this region (Table 2).

### Multiclimate Phytogeographic Region

The Multiclimate phytogeographic region is not distinctly associated with any specific biome or habitat (Table 1; Figures 1 and 3), although the climatic variables best predicting its distribution is exactly opposite to that of the Greater Arid region, i.e. milder and wetter (Table 2). The six lower level phytogeographic regions embedded within this main region are more distinct in terms of climate and environmental features.

#### Escarpment phytogeographic region

This region is distributed mainly along the length of the eastern and southern side of the Great Escarpment, being most concentrated at the Drakensberg range in KwaZulu-Natal province, bordering (and overlapping) Lesotho, and to a lesser extent, the Lebombo mountains on the border between Mpumalanga and the west of Swaziland (Figures 1 and 4). It is mainly covered by Grassland, with a smaller contribution by Savanna and other biomes (Table 1, Figure 1). It shows spatial congruence with the mistbelt on the eastern side of the escarpment, which is a cool, moist temperate region within the Grassland biome that is characteristically wetter than other grasslands (consistent with the regression model for this region, Table 2) and includes many small patches of natural forest. The Escarpment phytogeographic region is characterised by alien plant species that originate from cool, moist temperate regions such as northern Europe. This includes mainly temperate C_3_ grasses such as *Bromus catharticus, Poa annua*, *Poa pratensis*, *Holcus lanatus*, *Phalaris arundinacea*, and *Phalaris dilitatum*. The phytogeographic region forms a sharp border on the escarpment, especially at Lesotho and the KwaZulu-Natal Drakensberg, which is unsurprising because the alien species named probably require year round moisture, as found in the mistbelt, whereas the environmental conditions on the adjacent high-altitude Afroalpine grassland region is harsh, with cold, dry winters. Irrigated area and degraded area are also coincident with this region according to the regression model, suggesting a possible human influence (Table 2).

#### Northern phytogeographic region

This region is distributed mainly in the north of Namibia and Botswana, with some outliers in the north of South Africa (Figures 1 and 4). It is the most geographically dispersed of all clusters of grid cells that we reported as phytogeographic regions. It is characterised by tropical alien plants that do not tolerate cold conditions (e.g. frost), and includes tropical water plants such as *Salvinia molesta* and *Persicaria limbata*, and riverside plants such as *Mimosa pigra* and *Sesbania bispinosa*. This region appears to be found in relatively arid low altitude areas with water sources, such as the Okavango Delta and Kunene River on the northern boundaries of Namibia and Botswana, and seems to constitute the southern outliers of a tropical phytogeographic region with its core situated to the north of the study area. However, the paucity of data in this area precludes any further analysis and interpretation.

#### Agricultural phytogeographic region

This region is associated with several biomes and different rainfall patterns and does not appear to be a consistent, spatially unified phytogeographic region, except that it is associated with high mean maximum temperatures throughout its range (Tables 1 and 2). It is closely associated with the Escarpment phytogeographic region in certain areas, and continues along the escarpment to the south of the mistbelt where it is most concentrated in the summer rainfall region of the Eastern Cape province, bordering much of the coastline (Figures 1 and 4) [37]. However, the Agricultural region is also prominent in the Western Cape province, where it is concentrated in the centre of the Fynbos biome and winter rainfall zone, especially in the Breede River valley and Swartland areas (Figures 1 and 4) [37]. In addition, a substantial group of grid cells are concentrated in the summer rainfall interior of South Africa, in the Northern Cape and Free State provinces (Figures 1 and 4). The Agricultural region is characterised by temperate C_3_ alien grasses, such *Briza maxima*, *B. minor*, *Hordium murinum*, *Bromus diandrus*, *Vulpia myuros*, and *Phalaris minor*, and a few species such as *Poa annua* that are shared with the Escarpment region. These species require winter precipitation and are often planted for winter fodder and encouraged by irrigation to grow where there is naturally no winter rainfall, such as in the Northern Cape, Free State and Eastern Cape Provinces where irrigation is common along rivers. Further, agriculture reduces competition by clearing native vegetation, and changes soil nutrient input and edaphic features, and may thereby encourage pioneer alien grasses that are able to quickly colonise disturbed land [23]. These may be important factors for this phytogeographic region in the Swartland and Breede River Valley regions in the Western Cape Province, where the native vegetation had mostly been converted to agricultural land such as winter wheat fields and vineyards [54]. These observations suggest that human activities, such as agriculture and irrigation that artificially manipulate vegetation cover and edaphic and climatic factors, are important unifying features that link the various clusters of this phytogeographic region across the study area. This suggestion is not well supported by the regression model for this region, as cultivated and degraded area is not included in the model and irrigated area makes only a small positive contribution (Table 2). However, the small Max-rescaled R-square value of this model (9%, Table 2) suggests that the spatial distribution of this region is very likely determined by variables that have not been considered in the current study, which might be human-related variables. Further, as mentioned previously, perhaps a human influence on this phytogeographic region may be obscured at the coarse spatial resolution of this study [21]. For example, in the dataset used for the logistic regression model, irrigated area usually comprise less than 10% of the surface area of the grid cells in which it is present. Therefore, alien plant species that are facilitated by irrigation might be spatially associated with small patches of irrigated area within the quarter degree grid cells in which they were recorded. A finer spatial resolution might reveal this association more explicitly; nevertheless, irrigated area would remain an important factor in the overall phytogeographic region.

#### Western Cape phytogeographic region

This region is most concentrated in the Western Cape Province, but extends north into the Northern Cape Province and east into the Eastern Cape Province (Figures 1 and 4). It borders most of the coastline in the region covered and is mainly a temperate area with mild winters, including areas with relatively high winter and year-round rainfall and the Fynbos and Succulent Karoo biomes (Tables 1 and 2; Figures 1 and 4) [37], [54]. It is characterised by herbs that are weeds in agricultural areas and disturbed valleys and annuals that are strongly dependent on winter rain, such as *Hordium murinum*, *Briza maxima*, *B. minor*, *Phalaris minor*, *Vulpia myuros*, *V. bromoides, Fumaria muralis* and *Lolium rigidum*. Many accidentally introduced species characterise this region; however, although cultivated area is not particularly strongly associated with this region (Table 2), deliberate introduction also seemed to have had a great influence here, and the Agricultural and Western Cape phytogeographic regions share several C_3_ grass species.

#### Grassland phytogeographic region

This region mainly comprises the Grassland biome in the interior of South Africa north and west of the escarpment and bordering the Escarpment phytogeographic region, in the provinces North West, Gauteng, Mpumalanga, the west of KwaZulu-Natal, the northern edge of the Eastern Cape and most of Lesotho (Table 1; Figures 1 and 4) [37]. It also extends north into the Savanna biome of North West Province, Namibia and Botswana (Figures 1 and 4)[37]–[39]. The Grassland region is mainly characterised by cold, dry winters with frost and warm temperate summers with summer rainfall (see [55] and the regression model for this region, Table 2), and is characterised by herbs and grasses that are associated with agriculture, cultivation and abandoned agricultural fields, e.g. *Oenothera rosea*, *O. tetraptera*, *Salvia stenophylla*, *Medicago laciniata*, *Hibiscus trionum*, *Persicaria lapathifolia* and *Cirsium vulgare*. The first four species named most likely invade specifically moist areas within the Grassland biome, and *C. vulgare* is mainly associated with wetlands.

#### Savanna phytogeographic region

This region is mainly situated in warm frost-free Savanna biome areas of South Africa in the provinces North West, Limpopo, Mpumalanga, the east of KwaZulu-Natal, a small eastern part of the Northern Cape, and also most of Swaziland (Tables 1 and 2; Figures 1 and 4) [37]. It extends north into, and is spread over, the entire Botswana and Namibia, where it is the most prevalent phytogeographic region (Figures 1 and 4). It is also associated with the coast in KwaZulu-Natal Province and covers the Indian Ocean Coastal Belt biome and the adjacent Savanna biome where it is probably associated with the numerous dry river valleys (Table 1, Figures 1 and 4) [56]. It includes all kinds of Savanna habitat: from dry to moist, and from fertile to infertile [56]. The alien plant species characterising this phytogeographic region are mainly herbs that colonise disturbed areas and are found at roadsides (e.g. *Lantana camara*), although degraded area has a weak negative correlation with this region (Table 2). Temperate C_3_ grasses and species characterising the Greater Arid region are least likely to be found in the Savanna region (Table S2).

### Further Research and Implications for Management

The alien species that appeared to be most characteristic of the phytogeographic regions (Table S2) were generally relatively range-restricted invaders, a few of which were well-known harmful invaders (e.g. *Prosopis* spp. [10]), whereas most were currently of less concern. However, some of the species of less concern, such as the alien grasses that play an important role in several of the alien phytogeographic regions described, have the potential to become increasingly harmful in the future and warrant more research and management attention than they are currently given [16], [19], [24], [57], [58]. Unsurprisingly, alien species that have small ranges (only a few grid cells) did not influence the designation of phytogeographic regions. Conversely, the very widespread harmful invaders (e.g. *Acacia* spp., *Opuntia* spp. and *Pinus* spp. [10], [17]) individually covered many of the alien phytogeographic regions together, which would mean that these species did not have a strong influence on TWINSPAN in grouping grid cells into phytogeographic regions (abundance or dominance is not taken into account when a presence/absence dataset is used). The analysis methods of Richardson and colleagues [29] and Thuiller and colleagues [30], as described in the introduction section, are probably more suitable than the current study’s methods for revealing the spatial patterns of these widespread species.

As mentioned in the introduction, the presence of phytogeographic regions may be helpful in organising alien species management; however, the current study is mainly an explorative study, and the findings need to be refined before it will be useful for practical applications. Nevertheless, the findings provide a framework for further research, enabling further refinement of the spatial distributions and species compositions of the described phytogeographic regions (contingent on improved data at a finer spatial resolution) and more in-depth understanding of the environmental factors (natural and human-related) that determine each region. Future studies could explore the link between alien phytogeographic regions and the species traits that had predisposed the alien species to colonise those regions and exploit the available niche space. In particular, considering that alien phytogeographic regions formed over a much shorter time period (i.e. since the introduction of the characteristic species) than endemic phytogeographic regions [31], [32], were alien phytogeographic regions shaped in the introduced range by original alien species traits (i.e. those traits that had evolved in their original home range) or by rapid adaptation to new conditions (i.e. new or changed traits)?

It is likely that the spatial ranges of alien phytogeographic regions might shift in the near future as the spatial ranges of the characteristic species shift in response to changes in general climatic conditions (i.e. global climate change) as well as projected increases in human-induced microclimates and transformed habitats (e.g. irrigation and agriculture) that favour invasive alien plant species [23], [24], [27], [30], [59]. Similarly, climate change and local human activities are predicted to cause range shifts of plant species in general in the near future, thereby reorganising current plant assemblages to create new assemblages consisting mainly of plants with rapid colonisation abilities (a common characteristic of alien plant species) where the ranges of specialised native species have retreated, a process known as biotic homogenisation [6], [23], [24], [60], [61]. The alien phytogeograpic regions revealed in the current study might indicate some of the characteristics of future plant assemblages if such a scenario prevails. Correspondingly, this prediction also implies that short-term anthropogenic processes could influence the ranges and compositions of phytogeographic regions of endemic plant species [34] or genera [31], along with long term natural climatic and geological changes.

### Conclusions

We found that the study area could be partitioned into several ecologically interpretable phytogeographic regions. These phytogeographic regions primarily follow natural climatic, biome and habitat features, but are also influenced by anthropogenic modification and activities to varying degrees. Although this study is mainly explorative, our findings generate a suite of new hypotheses, and so open many possibilities for further research to refine and explain the spatial distributions and determinants of these phytogeographic regions. This is contingent on improved species presence data at a finer resolution. We suggest that, after appropriate further research, these phytogeographic regions could provide information benefitting the organisation of effective local management of currently or potentially harmful alien plant species. Further, we suggest that the characteristics (i.e. the species and the associated natural and anthropogenic factors) of these phytogeographic regions provide a glimpse of the likely floristic composition of regional plant assemblages of the future in a scenario where biotic homogenisation and range shifts have reorganised the current plant assemblages.

## Supporting Information

Table S1 **Alien plant species in each quarter-degree square for South Africa, Lesotho, Swaziland, Namibia and Botswana.** Naturalised alien plant species recorded in each quarter-degree square (QDS), derived from the National Herbarium’s, Pretoria Computerised Information System (PRECIS). PRECIS records are available online at http://posa.sanbi.org/, and the complete PRECIS dataset is available, on request, by contacting the data section of the National Herbarium at precis@sanbi.org.za.
(XLS)

Table S2 **The characteristic alien plant species of each phytogeographic region.** The phytogeographic regions of the current study were differentiated from one another and classified according to the alien plant species that were most characteristic of each particular region, i.e. that were more likely to occur in that particular region than in the rest of the study area. With the method used in the current study, the different regions could not overlap geographically but often shared characteristic species. Here we list, for each phytogeographic region, the species that occupied a greater proportion of a particular phytogeographic region than the rest of the study area (corrected for the sizes of the areas). We list only those species that occupied 5% or more of a region and were within the top twenty species, ranked according to the difference between the phytogeographic region and rest of the study area in percentage of grid cells occupied. The percentage of grid cells occupied by a species in a phytogeographic region is included in brackets.
(PDF)
