# Supplementary material for: Alien Phytogeographic Regions of Southern Africa: Numerical Classification, Possible Drivers, and Regional Threats
Source: PLoS One. 2012 May 4;7(5):e36269. doi: 10.1371/journal.pone.0036269 (PMC3344867; doi:10.1371/journal.pone.0036269)
Supplement: Table S2 — The characteristic alien plant species of each phytogeographic region. The phytogeographic regions of the current study were differentiated from one another and classified according to the alien plant species that were most characteristic of each particular region, i.e. that were more likely to occur in that particular region than in the rest of the study area. With the method used in the current study, the different regions could not overlap geographically but often shared characteristic species. Here we list, for each phytogeographic region, the species that occupied a greater proportion of a particular phytogeographic region than the rest of the study area (corrected for the sizes of the areas). We list only those species that occupied 5% or more of a region and were within the top twenty species, ranked according to the difference between the phytogeographic region and rest of the study area in percentage of grid cells occupied. The percentage of grid cells occupied by a species in a phytogeographic region is included in brackets. (PDF) [file pone.0036269.s002.pdf]

Table S2. The characteristic alien plant species of each phytogeographic region.

The phytogeographic regions of the current study were differentiated from one another and classified according to the alien plant species that were most characteristic of each particular region, i.e. that were more likely to occur in that particular region than in the rest of the study area. With the method used in the current study, the different regions could not overlap geographically but often shared characteristic species. Here we list, for each phytogeographic region, the species that occupied a greater proportion of a particular phytogeographic region than the rest of the study area (corrected for the sizes of the areas). We list only those species that occupied 5% or more of a region and were within the top twenty species, ranked according to the difference between the phytogeographic region and rest of the study area in percentage of grid cells occupied. The percentage of grid cells occupied by a species in a phytogeographic region is included in brackets.

Rank	1. Greater Arid	1.1 Arid	1.2 Orange River	2. Multiclimate
1	*Atriplex lindleyi* (23)	*Atriplex lindleyi* (32)	*Prosopis glandulosa* (59)	*Hibiscus trionum* (13)
2	*Prosopis glandulosa* (20)	*Salsola kali* (27)	*Prosopis velutina* (52)	*Achyranthes aspera* (12)
3	*Salsola kali* (21)	*Coronopus integrifolius* (26)	*Persicaria limbata* (10)	*Persicaria lapathifolia* (9)
4	*Coronopus integrifolius* (19)	*Polypogon monspeliensis* (18)	*Prosopis chilensis* (6)	*Rumex acetosella* (7)
5	*Prosopis velutina* (16)	*Verbesina encelioides* (11)		*Oenothera rosea* (7)
6	*Verbesina encelioides* (9)	*Atriplex nummularia* (9)		*Paspalum dilatatum* (9)
7	*Polypogon monspeliensis* (14)	*Atriplex eardleyae* (5)		*Lantana camara* (6)
8	*Atriplex nummularia* (7)	*Juncus bufonius* (6)		*Verbena bonariensis* (7)
9		*Puccinellia fasciculata* (5)		*Amaranthus hybridus* (6)
10		*Chenopodium murale* (7)		*Digitaria sanguinalis* (7)
11		*Lophochloa pumila* (5)		*Oxalis corniculata* (6)
12				*Gomphrena celosioides* (6)
13				*Schkuhria pinnata* (7)
14				*Hypochaeris radicata* (5)
15				*Oenothera tetraptera* (5)
16				*Poa annua* (5)
17				*Bromus catharticus* (7)
18				*Bidens pilosa* (5)

Table S2 continues

Rank	2.1 Mistbelt	2.2 Northern	2.3 Agricultural	2.4 Western Cape
1	*Verbena bonariensis* (16)	*Mimosa pigra* (7)	*Polypogon monspeliensis* (19)	*Polypogon monspeliensis* (29)
2	*Rumex acetosella* (15)	*Cocculus hirsutus* (7)	*Briza maxima* (11)	*Hordeum murinum* (25)
3	*Oenothera rosea* (13)	*Senna obtusifolia* (5)	*Paspalum dilatatum* (16)	*Spergularia media* (20)
4	*Paspalum dilatatum* (15)	*Glinus lotoides* (6)	*Lolium multiflorum* (10)	*Briza maxima* (19)
5	*Persicaria lapathifolia* (15)	*Salvinia molesta* (5)	*Hypochaeris radicata* (12)	*Aira cupaniana* (15)
6	*Phalaris arundinacea* (8)	*Datura innoxia* (5)	*Aira cupaniana* (10)	*Bromus diandrus* (14)
7	*Holcus lanatus* (7)	*Digitaria sanguinalis* (10)	*Poa annua* (12)	*Vulpia myuros* (14)
8	*Lantana camara* (12)	*Eclipta prostrata* (5)	*Rumex acetosella* (13)	*Brachypodium distachyon* (12)
9	*Cassytha filiformis* (10)	*Acanthospermum hispidum* (5)	*Vulpia bromoides* (9)	*Vulpia bromoides* (13)
10	*Poa annua* (10)	*Flaveria bidentis* (7)	*Digitaria sanguinalis* (12)	*Lolium rigidum* (12)
11	*Poa pratensis* (7)	*Phyla nodiflora* (5)	*Hordeum murinum* (11)	*Anagallis arvensis* (12)
12	*Polygonum aviculare* (10)	*Persicaria limbata* (5)	*Briza minor* (8)	*Polypogon viridis* (12)
13	*Solanum nigrum* (11)	*Sesbania bispinosa* (6)	*Bromus diandrus* (8)	*Solanum americanum* (10)
14	*Senna septemtrionalis* (7)	*Ricinus communis* (6)	*Silene gallica* (7)	*Fumaria muralis* (11)
15	*Acanthospermum australe* (7)	*Senna occidentalis* (5)	*Juncus bufonius* (6)	*Briza minor* (11)
16	*Bromus catharticus* (12)		*Phytolacca octandra* (8)	*Phalaris minor* (11)
17	*Rubus cuneifolius* (7)		*Vulpia myuros* (8)	*Medicago polymorpha* (11)
18	*Bidens pilosa* (9)		*Bromus catharticus* (11)	*Bromus hordeaceus* (10)
19	*Ageratum conyzoides* (7)		*Phalaris minor* (7)	*Silene gallica* (11)
20	*Echium vulgare* (6)		*Medicago polymorpha* (7)	*Lotus subbiflorus* (10)

Table S2 continues

Rank	2.5 Grassland	2.6 Savanna
1	*Oenothera rosea* (21)	*Achyranthes aspera* (23)
2	*Medicago laciniata* (19)	*Alternanthera sessilis* (9)
3	*Oenothera tetraptera* (19)	*Sesbania bispinosa* (9)
4	*Hibiscus trionum* (17)	*Gomphrena celosioides* (12)
5	*Paspalum dilatatum* (25)	*Senna occidentalis* (8)
6	*Rumex acetosella* (21)	*Richardia scabra* (8)
7	*Persicaria lapathifolia* (18)	*Euphorbia indica* (8)
8	*Bromus catharticus* (20)	*Flaveria bidentis* (9)
9	*Amaranthus hybridus* (17)	*Lantana camara* (11)
10	*Cirsium vulgare* (16)	*Boerhavia diffusa* (7)
11	*Chenopodium schraderianum* (13)	*Ageratum conyzoides* (8)
12	*Oxalis corniculata* (12)	*Cocculus hirsutus* (7)
13	*Polygonum aviculare* (15)	*Hybanthus enneaspermus* (7)
14	*Cuscuta campestris* (13)	*Polycarpaea corymbosa* (7)
15	*Rumex crispus* (13)	*Acanthospermum hispidum* (7)
16	*Pseudognaphalium luteoalbum* (12)	*Corchorus trilocularis* (7)
17	*Digitaria sanguinalis* (17)	*Hibiscus trionum* (18)
18	*Myosotis sylvatica* (14)	*Phyla nodiflora* (7)
19	*Schkuhria pinnata* (10)	*Ethulia conyzoides* (7)
20	*Physalis angulata* (9)	*Richardia brasiliensis* (10)
